# A Seizure and Hemiplegia following Contrast Exposure: Understanding Contrast-Induced Encephalopathy

**DOI:** 10.1155/2018/9278526

**Published:** 2018-03-04

**Authors:** Bhargavi Donepudi, Steven Trottier

**Affiliations:** ^1^Department of Internal Medicine, Mercy Hospital, St. Louis, MO, USA; ^2^Department of Critical Care, Mercy Hospital, St. Louis, MO, USA

## Abstract

Contrast-induced encephalopathy is a rare, reversible phenomenon known to occur after intravenous or intra-arterial contrast exposure. This report describes a case involving a 73-year-old female admitted for an elective thoracic aortic aneurysm repair. During the procedure, a large volume of nonionic iodinated contrast was necessary for arteriography. Postoperatively, the patient developed seizure activity followed by left-sided hemiplegia. Computed tomography (CT) of the brain without contrast and magnetic resonance imaging (MRI) were negative for acute stroke but did show residual contrast surrounding the brain. Antiepileptic medications were administered with resolution of the seizure activity. The patient was treated with supportive management and improved to baseline over the next seven days. This case demonstrates a rare, nonionic iodinated contrast-induced encephalopathy with seizure activity and transient hemiplegia. The unique imaging findings differentiate it from other neurologic conditions.

## 1. Introduction

The most common known side effects of iodinated contrast material include hypersensitivity reactions and contrast-induced nephropathy. Very few case reports in the literature describe contrast-induced encephalopathy (CIE). The clinical spectrum of CIE ranges from confusion to cortical blindness, seizures, hemiplegia, and coma [1, 2]. CT of the brain without contrast and MRI of the brain with an apparent diffusion coefficient (ADC) map differentiate CIE from acute stroke, subarachnoid hemorrhage (SAH), and posterior reversible encephalopathy (PRES) [2–5].

## 2. Case

**Figure 1:**
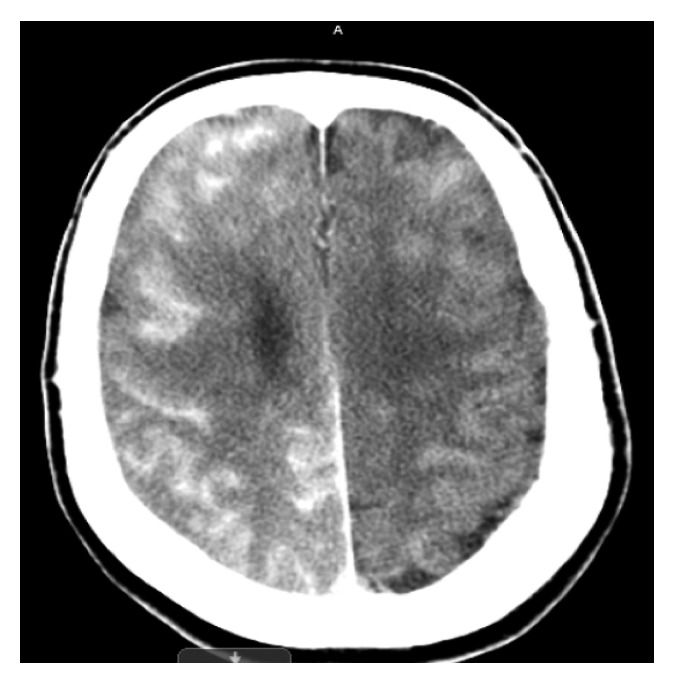
CT brain showing increased hyperdensity and enhancement of right cortex.

**Figure 2:**
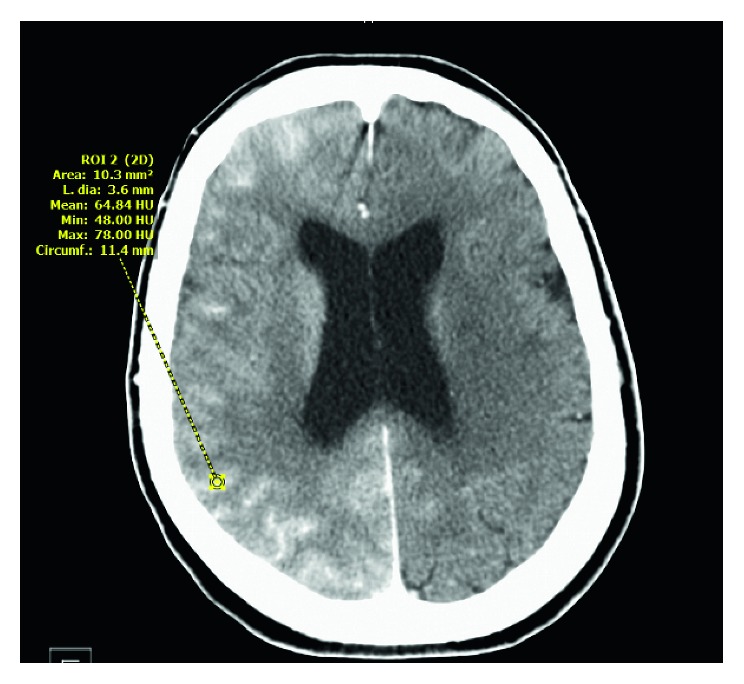
Measurement of Hounsfield units (HU) which differentiate blood (30–45 HU) from contrast material (80–160). It shows a mean of 64 HU.

**Figure 3:**
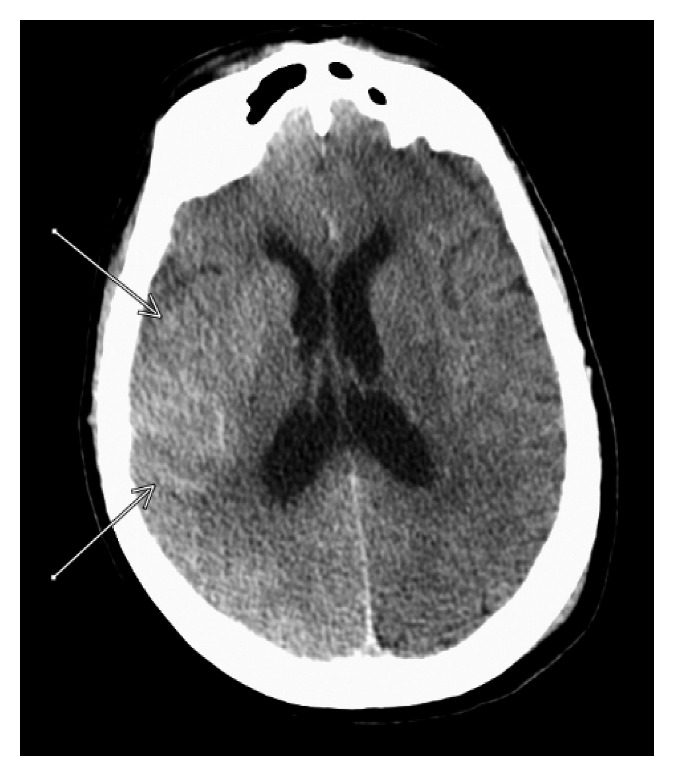
CT of the brain on POD one showed decreased hyperintensity on the right side of the cortex compared to the CT of the brain immediately post-op (Figure 1).

**Figure 4:**
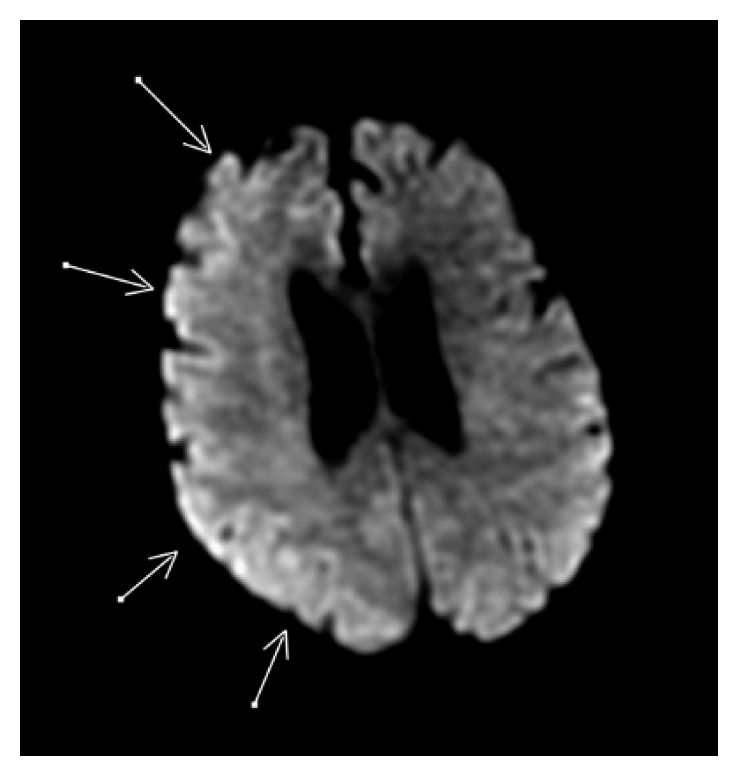
MRI with DWI showing diffuse involvement of the frontoparietal region.

A 73-year-old female with a past medical history of hypertension, hyperlipidemia, chronic kidney disease (CKD) stage 3 (admission creatinine 1.38 mg/dl and glomerular filtration rate (GFR) 37 ml/min), myocardial infarction status after coronary stent placement, abdominal aortic aneurysm (AAA), and peripheral vascular disease status after left iliofemoral and femoral popliteal bypass grafts and right carotid stent placement was admitted for elective thoracic aortic aneurysm (TAA) repair. Computed tomography angiography (CTA) chest/abdomen/pelvis with contrast done prior to admission revealed increase in the size of thoracoabdominal aortic aneurysm which measured 4.3 × 5.2 cm with the largest AP diameter of 6.1 cm in the AP axis. The patient's neurological status on admission was alert and oriented. She had normal motor strength with sensation intact in all extremities. She had no history of stroke or seizure disorder. Preoperatively, a lumbar drain was placed for spinal cord protection. The goal of the lumbar drain was to keep the spinal cord perfusion pressure greater than 60 mmHg and the spinal pressure less than 10 mmHg. The patient was taken to the operating room (OR) for the placement of thoracic aortic (Zenith Alpha thoracic proximal component aortic stent graft measuring 30 × 201 mm, Zenith TX2 TAA proximal aortic extension measuring 30 × 80 mm, and Zenith Alpha thoracic endovascular graft to the descending aorta measuring 34 × 112 mm) and infrarenal aortic endograft, right iliac artery stent (VIABAHN Hep, 10 mm × 10 cm), and bilateral iliofemoral artery bypass grafts (Hemashield Gold vascular graft, 10 mm × 30 cm). A total of 248 ml of iodixanol (Visipaque) containing 320 mg·iodine/ml intra-arterial injection was administered for aortic arch and thoracic aortic arteriogram to evaluate anatomy and blood flow and for endoleak assessment. The entire procedure lasted for about five hours. Throughout the procedure, a total of 25,000 units of heparin was used to heparinize the patient, and activated clotting time (ACT) ranged between 143 and 219 seconds. After the procedure, the patient was noted to be hypothermic with a temperature of 33.6 degrees Celsius. She was admitted to the intensive care unit (ICU) on a postoperative propofol infusion and received mechanical ventilator support. Norepinephrine was administered to achieve a mean arterial pressure of 100–110 mmHg. Vital signs upon arrival to the ICU were as follows: BP 166/76, RR 12, and HR 63. An hour after the ICU admission, the propofol infusion was titrated down at which time the patient was noted to have a focal motor seizure in her left leg, arm, and face lasting about 20 minutes despite the administration of 4 mg lorazepam and increasing her propofol. Following the seizure activity, CT of the brain without contrast was performed revealing an abnormal hyperdensity and extensive enhancement of the right cortex, subarachnoid space, and basal ganglia (Figures 1 and 2). CSF fluid analysis was not performed. The patient was started on levetiracetam 1000 mg twice daily. A 24-hour continuous electroencephalogram was negative for seizure activity. CT of the brain 24 hours later (Figure 3) showed decreased enhancement on the right side of the brain compared to initial CT scan. Neurologic examination without sedation on postoperative (POD) day one demonstrated a left-sided hemiplegia. The patient sustained contrast-induced nephrotoxicity which may have further contributed to contrast retention. Intravenous fluids were continued with very minimal improvement in her kidney function (with creatinine peaked at 2.37 mg/dl with a GFR of 20 on the second POD). Lumbar drain was removed on POD two. Follow-up MRI on POD two was negative for focal lesions to indicate infarct. Some asymmetry on the FLAIR imaging and the diffusion-weighted sequence that was fairly diffuse involving the cortex especially over the frontoparietal region was noted (Figure 4). On POD four, the patient was extubated and started to regain movement on her left side. By POD seven, the patient regained 4/5 strength. The patient was seen in follow-up two months after the hospitalization and had returned to her baseline neurologic function.

## 3. Discussion

Contrast-induced encephalopathy is an acute reversible encephalopathy occurring within minutes to hours after a known insult with intravascular or intrathecal contrast injections [1, 2, 4–6]. Possible risk factors for CIE include chronic hypertension with impaired cerebral auto regulation, CKD/end-stage renal disease, diabetes, previous reaction to contrast material, intracranial pathology, direct injection into the cerebral circulation, and a large volume of injected contrast. However, CIE may also occur following low volumes of contrast, as low as 50 ml [1, 2, 4]. This patient had a history of chronic HTN, CKD3, and large amount of contrast (248 ml) administration for the arteriogram.

CIE may mimic acute stroke and SAH. The most commonly described symptoms include headache, vomiting, agitation, cortical blindness, hemiplegia, seizures, confusion, transient global amnesia, and dysarthria-aphasia. Myoclonus and coma may occur rarely. CIE symptoms typically resolve within 7 days [1–6].

### 3.1. Pathophysiology

The blood-brain barrier (BBB) is a selectively semipermeable membrane preventing many materials from entering into the brain parenchyma. Normally, the BBB does not allow iodinated contrast molecules to enter the brain. However, when the BBB is disrupted, intravenous contrast molecules may enter the CNS leading to direct chemical neurotoxicity. The osmotic property of the extravasated contrast pulls fluid into the brain leading to cerebral edema. One of the current theories leading to the BBB disruption is that hyperosmolar/ionic contrast agents remove fluid from endothelial cells thus causing them to shrink thus widening the tight junctions [1–6]. However, a few case reports involving iso-osmolar contrast agents and CIE have been published [1–6]. The current case report describes CIE occurring after exposure to 248 ml of the iso-osmolar contrast agent iodixanol for aortic arteriogram. When given in large doses, iso-osmolar contrast agent may lead to BBB disruption by having direct toxic effect on endothelial cells. This effect may be compounded when patient has uncontrolled hypertension and CKD.

Iodixanol is a nonionic dimeric agent with an osmolality of 290 similar to the blood. This contrast agent distributes freely and does not bind to the plasma proteins making it dialyzable. The elimination half-life is nearly two hours in patients with normal kidney function and up to 23 hours with kidney dysfunction [1].

The differential diagnosis for CIE includes ischemic stroke, SAH, Todd's paralysis, and PRES.

### 3.2. Diagnosis

Neuroimaging paired with known exposure to a contrast agent is the key for suspecting the diagnosis of CIE. Immediate CT scan of the brain to exclude ischemic and hemorrhagic stroke (including SAH) is required.

The CT findings in CIE range from diffuse cortical and subcortical enhancement to focal hyperdense lesions, enhanced cerebral sulci, cerebral edema, and enhancement in subarachnoid space mimicking SAH. Measuring the Hounsfield units (HU) can help differentiate SAH from CIE. Blood usually measures at 30–45 HU, and contrast usually measures at 80–160 HU [2, 4, 5].

Cerebral edema (vasogenic and cytotoxic) can be seen in multiple conditions including CIE, stroke, PRES, and tumors. Cytotoxic cerebral edema can be distinguished from vasogenic edema on DWI images. Cytotoxic cerebral edema has restricted diffusion, whereas vasogenic has normal or increased diffusion on DWI. Acute ischemic stroke can be visualized as area of hyperintensity with restricted diffusion on DWI images which matches with hypointense area on apparent diffusion coefficient (ADC) map [3, 5]. In CIE, MRI findings include hyperintense areas on all T2, DWI, and flair images with no change in ADC map [2, 5]. Another important condition to distinguish is posterior reversible encephalopathy syndrome (PRES). Posterior reversible encephalopathy syndrome has many similarities with CIE including risk factors, clinical manifestations, and imaging findings. However, PRES usually has symmetrical bilateral involvement with predilection to regions supplied by posterior circulation. Involvement of the subcortical white matter is more prominent in PRES and does not typically involve subarachnoid spaces. The involved area of brain parenchyma shows increased ADC values in contrast to no change in ADC for CIE [5, 7].

In perfusion-weighted imaging, normal blood flow is seen in CIE, whereas decreased flow is detected in ischemic stroke and increased flow is seen to an area of ictal focus in seizures [3].

The lumbar drain utilized during this case requires comment. Lumbar drains are frequently used to decrease the risk of spinal cord injury during thoracic aortic aneurysm repairs. The goal of using the lumbar drain is to maintain the spinal perfusion pressure and avoid spinal cord ischemia. The complications associated with lumbar drains include headache, bleeding, infection, retained catheter fragments, hematoma, radiculopathy, and excessive spinal fluid drainage. Excessive spinal fluid drainage can result in intracranial hemorrhage and cerebral hypotension leading to cerebral vein thrombosis. Intracranial hemorrhage and cerebral vein thrombosis caused by excessive spinal fluid drainage can result in seizure activity. This patient did not have excessive spinal fluid drainage. In addition, the anesthesia team confirmed that the patient did not receive intrathecal contrast injection. Direct intrathecal contrast injection is associated with seizure activity [8–10].

In conclusion, CIE is typically a reversible condition that should be considered in the differential diagnosis of an acute central nervous system change following exposure to an intravascular or intrathecal contrast agent. CIE should be managed with supportive treatment including intravenous fluids to facilitate contrast excretion and antiepileptic medications for seizure prophylaxis. If patient has ESRD, hemodialysis should be considered to remove the contrast agent [1]. Mannitol and corticosteroids have been administered to patients with CIE and significant cerebral edema; however, these agents have not been shown to improve outcomes [4]. With supportive management, recovery is always near complete within one week.
